# Maternal protein-energy malnutrition during early pregnancy in sheep impacts the fetal ornithine cycle to reduce fetal kidney microvascular development

**DOI:** 10.1096/fj.14-255364

**Published:** 2014-11

**Authors:** Louise J. Dunford, Kevin D. Sinclair, Wing Y. Kwong, Craig Sturrock, Bethan L. Clifford, Tom C. Giles, David S. Gardner

**Affiliations:** * School of Veterinary Medicine and Science,; † School of Biosciences, and; ‡ Advanced Data Analysis Centre, University of Nottingham, Sutton Bonington Campus, Loughborough, UK

**Keywords:** polyamines, renal

## Abstract

This paper identifies a common nutritional pathway relating maternal through to fetal protein-energy malnutrition (PEM) and compromised fetal kidney development. Thirty-one twin-bearing sheep were fed either a control (*n*=15) or low-protein diet (*n*=16, 17 *vs.* 8.7 g crude protein/MJ metabolizable energy) from d 0 to 65 gestation (term, ∼145 d). Effects on the maternal and fetal nutritional environment were characterized by sampling blood and amniotic fluid. Kidney development was characterized by histology, immunohistochemistry, vascular corrosion casts, and molecular biology. PEM had little measureable effect on maternal and fetal macronutrient balance (glucose, total protein, total amino acids, and lactate were unaffected) or on fetal growth. PEM decreased maternal and fetal urea concentration, which blunted fetal ornithine availability and affected fetal hepatic polyamine production. For the first time in a large animal model, we associated these nutritional effects with reduced micro- but not macrovascular development in the fetal kidney. Maternal PEM specifically impacts the fetal ornithine cycle, affecting cellular polyamine metabolism and microvascular development of the fetal kidney, effects that likely underpin programming of kidney development and function by a maternal low protein diet.—Dunford, L. J., Sinclair, K. D., Kwong, W. Y., Sturrock, C., Clifford, B. L., Giles, T. C., Gardner, D. S.. Maternal protein-energy malnutrition during early pregnancy in sheep impacts the fetal ornithine cycle to reduce fetal kidney microvascular development.

The World Health Organization estimates that 1.1 million children in the United States (0.8% of sampled population) suffer from protein-energy malnutrition (PEM; ref. 1). Globally, PEM is prevalent (8% incidence), contributing toward the estimated 32 million babies born small for gestational age each year, which significantly increases rates of childhood mortality (2). Humans evolved eating a seasonal diet high in plant-based foodstuffs (fruits, vegetables, seeds, and nuts) supplemented with lean, animal-derived protein (3). Such a diet has a relatively high protein-energy density and was replete with essential micronutrients, vitamins, and minerals. While the rates of overweight and obesity testify to the quantity of nutrition not being limiting in the majority of Western countries, often the nutritional quality remains poor, with malnutrition (*e.g.*, PEM) coexisting with obesity in many countries and even within families (2, 4, 5). Malnutrition during pregnancy has negative outcomes that extend beyond the progeny (6–9); more recently, this hypothesis has become known as the “developmental origins of health and disease” and was a paradigm shift in our understanding of the relationship between nutrition and reproductive biology (10–14). Animal models have since filled the knowledge gap between maternal diet and adult disease with some mechanistic detail (15, 16). However, while there is a plethora of information for expectant mothers on what should constitute an appropriate maternal diet (*e.g.*, in the United Kingdom; http://www.nhs.uk/start4life), and much is known about fetal nutrition, endocrinology, and growth *per se* (17–19), a mechanistic pathway from maternal malnutrition, potentially through fetal malnutrition, to a specified disease outcome has not been elucidated.

Low maternal intake of protein is associated with intrauterine growth-restricted infants that become susceptible to chronic kidney and/or cardiovascular disease (20–22). With this in mind, we developed a large animal model of maternal PEM using sheep (23). The sheep is particularly suitable for such an experiment, having a long history of use for fetal physiological and developmental research (24) and nutritional requirements that are well characterized (7, 25). A nutritionally unbalanced maternal diet, with a decreased protein:energy ratio as opposed to a balanced reduction in energy availability, has greater significance for early fetal development because maternal metabolism is required to adjust substrate cycles to accommodate the lack of amino acids rather than simply “buffer” an energy deficit. The embryo (26) and subsequently the fetus (18) have specific requirements for particular patterns of substrate (glucose, amino acids, lactate) to enable optimal growth, which exponentially decline as gestation proceeds (27, 28). A high relative rate of growth (*i.e.*, during the embryonic rather than fetal period) therefore demands a highly specific pattern of substrate (particularly of amino acids) and metabolic intermediates for ordered development of cells, tissues, and organs. Thus, the quality of fetal nutrition is most important early in gestation.

A number of studies using differing animal models have shown that maternal protein or energy malnutrition can affect the quantity (*i.e.*, global reduction in fetal amino acid availability) and/or quality (*i.e.*, no change in total concentration but reduction in availability of specific amino acids) of the fetal nutritional milieu (29–33). On occasion, no effect (*i.e.*, no reduction in total or individual concentrations of amino acids in fetal plasma) has been reported (31, 34). This is likely due to differences in experimental design, the animal model, degree of maternal undernutrition, timing of sampling (*cf.* gestational age), and methodological techniques. With respect to the kidney, maternal PEM has been consistently shown to detrimentally affect fetal kidney development and later function, especially if instigated during the period of metanephrogenesis in various species (for reviews, see refs. 35, 36). In nonhuman primates, global undernutrition alters the fetal kidney transcriptome (37, 38), but no renal phenotype in the resultant adults has yet been described in this model. Hence, to our knowledge, no study has related maternal PEM to a pattern of nutrients in the fetal compartment that may account, in part, for physiological programming of fetal kidney development and function in later life. In this study, we were interested in how maternal PEM may influence nutrient patterns in the fetal compartment, but we were specifically interested in effects on the fetal ornithine cycle: PEM is likely to reduce maternal plasma urea through reduced protein turnover, the molar mass of urea (60 g/mol) is such that it easily crosses the placental barrier, and the urea/ornithine cycle delivers considerable substrate for polyamine synthesis, an important precursor of cell proliferation. Furthermore, investigation of prenatal factors that can negatively affect kidney development is of particular merit: the mammalian kidney has acquired its full complement of nephrons at, or shortly after, birth (39, 40), a limited capacity for repair after injury (41), and no clearly defined stem cell niche to replace lost functional units such as glomeruli (42). Hence, the kidney, much like the ovary, accommodates by having a large reserve functional capacity (*i.e.*, ∼70% loss of nephrons before clinical symptoms present) which buffers the inevitable and gradual loss of functional units with time. For the kidney, like the ovary, any residual deficit from birth represents a lifetime loss and may precipitate an exacerbated, age-related functional decline.

We have previously characterized an anatomically and physiologically compromised kidney in adult sheep that experienced maternal PEM during early gestation coupled with a superimposed obesogenic environment as adults (23). Here we have extended this model to consider the effects that maternal PEM has on the nutritional status of the mother and her fetus. In doing so, we were able to identify an altered pattern of nutrients that associates with compromised fetal kidney microvascular development. In addition, with sufficient numbers of male and female fetuses assigned to treatment groups, we were able to consider potential sex-specific interactions with maternal diet; an important consideration for scientific studies (43) and one that appears to underpin developmental origins of health and disease, regardless of the model organism (44).

## MATERIALS AND METHODS

### Ethical approval

All procedures were performed in accordance with the UK Animals (Scientific Procedures) Act, 1986 (Amended Regulations 2013), and were approved by the Animal Welfare and Ethical Review Board of the University of Nottingham. In determining sample size for the study, we considered variation in fetal amino acid concentrations to be the main outcome of interest. With respect to plasma ornithine, the study had 80% power (α, set at 0.05) to detect a 160 μM difference in the fetal plasma concentration of ornithine, analyzed in a split-plot design with maternal diet as the whole plot (variance, 26,310) and fetal sex as the subplot (variance, 27,504) and with *n* = 15 replicates/group.

### Animals and experimental design

Thirty-one pregnant Scottish Blackface ewes carrying twins were randomly allocated to groups fed either a control protein (CP) diet (*n*=15) providing adequate protein from d 0 to 65 gestation (term ∼147 d), or a protein-restricted low-protein (LP) diet from d 0 to 65 gestation (*n*=16). On an as-fed basis, the diets were isocaloric, with the effective level of protein restriction being 8.7 *vs.* 17 g crude protein/MJ metabolizable energy. These levels are in line with allowances recommended by the UK Agricultural Research Council (45) and the Agricultural and Food Research Council (46). Ewes were fed the concentrate ration as 2 even meals at 9:00 AM and 4:00 PM, with 100 g hay offered in the morning only to maintain rumen function. Ewes were weighed and body condition was scored [arbitrary units, 1 (emaciated) to 5 (obese) scale; ref. 47 to crudely assess their response to the diets. Blood samples were withdrawn by venepuncture prior to mating (baseline, nonpregnant) and subsequently at d 28 and 65 of pregnancy. Blood was withdrawn into K^+^-EDTA tubes and centrifuged at 3000 rpm (800 *g*) for 10 min; the resulting plasma was taken and stored at −20°C until further analysis. Ewes received their last feed at 4:00 PM on d 64 of gestation. At 9:00 AM on d 65 of gestation, the sheep were euthanized by barbiturate overdose (Dolethal; 150 mg/kg; Vetoquinol, Buckingham, UK), and the intact uteroplacental unit with twin fetuses was retrieved. In <1 min after euthanasia, amniotic fluid was sampled (10 ml) from each fetus and stored at −20°C. Subsequently, a fetal blood sample (5 ml) was withdrawn from an umbilical artery of each twin fetus and handled and stored as described above. One twin from each pair was semirandomly assigned (to balance the distribution of sex within treatment group) to undergo a vascular corrosion cast procedure (control, *n*=6 males and *n*=8 females; low protein, *n*=8 males and *n*=7 females) or a tissue collection procedure (control, *n*=8 males and *n*=8 females; low protein, *n*=9 males and *n*=8 females) in which selected tissues were either snap frozen in liquid nitrogen (*e.g.*, the right kidney) and stored at −80°C or fixed in 4% w/v paraformaldehyde (18–24 h at 4°C), rinsed in 0.02 M PBS for 24 h, and finally transferred and stored long term in 70% ethanol at room temperature. Due to occasional experimental difficulties, not all measurements were possible on all samples; hence the appropriate experimental *n* for comparison is indicated in individual figures and tables.

### Vascular corrosion casts

One fetus from each ewe was corrosion cast using 7 ml of resin from Batson's No. 17 Plastic Replica and Corrosion Kit (Polysciences, Inc., Warrington, PA, USA), injected *via* the umbilical artery in a retrograde direction in accordance with the manufacturer's protocol. After perfusion, the resin-cast whole fetus was allowed to set on ice for 2–3 h, followed by immersion in maceration solution (20% potassium hydroxide) for 12–18 h at 50°C to remove the surrounding soft tissue. Thereafter, the vascular cast was rinsed, dried, and stored at room temperature. The surface area and volume of the resin casts of each fetal kidney were quantified using a Nanotom high-resolution computed tomography scanner (GE Sensing and Inspection Technologies, Wunstorf, Germany). X-ray slices were taken every 40 μm throughout the cast, averaging 2000 slices/fetus. Using Nanotom software, these images were stacked and reconstructed in 3D (see Supplemental Movie S1) to provide quantitative volumetric data and to calculate the surface area of each cast.

### Metabolite analysis

Plasma samples were analyzed for glucose, nonesterified fatty acids (NEFAs), triglycerides, albumin, total protein, urea, lactate and β-hydroxybutyrate using a Randox Rx Imola autoanalyser (Randox Laboratories, Crumlin, UK), as described previously (48). The osmolality of maternal and amniotic samples was determined using a cryoscopic osmometer (Osmomat 030; Gonotec, Berlin, Germany). The system was calibrated against deionized H_2_O (0 Osmol) and a standard osmolar solution (300 mOsmol/kg H_2_O; Gonotec). Analysis of amino acids in biofluids, after derivatization, was according to the manufacturer's instructions (EZ:faast Amino Acid Kit; Phenomenex, Macclesfield, UK) and as described previously (48). Amino acid standards were prepared using the same method and were run every 15 samples to check for consistency.

### Analysis of fetal liver polyamines

Polyamines were determined based on the method of Ekegren & Gomes-Trolin (49). In brief, a 200 mg sample of fetal liver was homogenized on ice in HPLC-grade H_2_O, and an aliquot was used for determining the protein content (Bio-Rad DC kit). An internal standard (20 μl of 60 μM 1,6-DAH) was added to the homogenate (700 μg protein), and the sample was deproteinized using 4 M perchloric acid. The supernatant was adjusted to pH 9.0 using 4 M KOH and derivatized using 0.2 M borate buffer (pH 9.0) and 0.01 M FMOC reagent (in acetone). The reaction was stopped using 240 μl 0.04 M glycine. Twenty microliters of 10× diluted sample was injected into a Agilent 1000 HPLC system onto a Zorbax column C18 (250×4.6 mm ID, 5 μm; Agilent Technologies, Stockport, UK) protected by a 5 μm guard column maintained at 40°C. The fluorescent detector excitation and emission wavelengths were set at 264 and 310 nm, respectively. Mobile phase A was 0.05 M sodium acetate (pH 4.2), acetonitrile, 80/20 (v/v), and mobile phase B was 0.05 M sodium acetate (pH 4.2), acetonitrile, 5/95 (v/v). Unknown samples were interpolated from a standard curve (0–12 nM) with a lower limit of detection at 0.1 nM.

### Renal immunohistochemistry (IHC)

Renal sections were analyzed for the abundance of vascular endothelial growth factor A (VEGFA; SC-152; Santa Cruz Biotechnology, Santa Cruz, CA, USA) using a biotinylated ABC kit (Vector Laboratories, Peterborough, UK) and cluster of differentiation factor 34 (CD34; ab81289; Abcam, Cambridge, UK) using a Leica Bond-Max automated system (Leica Microsystems, Wetzlar, Germany). For VEGFA, the primary antibody was diluted 1:200 in PBS and 0.05% Tween and incubated overnight at 4°C. For CD34, the primary antibody was diluted 1:1000 in Bond Primary Antibody Diluent (Leica) and incubated for 30 min at room temperature. Heat-mediated antigen retrieval was carried out for 10 min using EDTA (pH 9). Negative controls were included during each IHC run by omission of primary antibody and using a rabbit IgG control (Vector Laboratories). For both proteins, adult ovine kidney was used as a positive control.

### Quantitative polymerase chain reaction (qPCR)

Total RNA was extracted using a PureLink Mini Kit (Invitrogen, Paisley, UK), and cDNA was synthesized using an Omniscript reverse transcriptase kit (Qiagen, Crawley, UK). Primers were designed using U.S. National Center for Biotechnology Information (NCBI) Primer-BLAST (NCBI, Bethesda, MD, USA; **Table 1**) and purchased from Eurofins MWG (Ebersberg, Germany). qPCR was performed using a Roche SYBR Green kit (Roche Diagnostics, Burgess Hill, UK) on a Roche Lightcycler 480 (Roche, Basel, Switzerland). Melt curves were used to confirm reaction specificity, and *cyclophilin*, β-*actin*, and *GAPDH* were used as housekeeping genes. mRNA quantities were normalized to housekeeping genes using Roche Lightcycler 480 advanced relative quantification software.

**Table 1.** Primer details for quantitative PCR

Primer	Sequence, 5′ to 3′	*T*_A_ (°C)
Angiopoietin 1	F: GGGTCACACTGGGACAGCAGG	58
	R: TGGGCCACAGGCATCAAACCA	
β-Actin	F: TGTGCGTGACATCAAGGAGAA	55
	R: CGCAGTGGCCATCTCCTG	
CDH11	F: ATCTCCCCCACAGAAATCGC	54
	R: CGGCCTCTTCTTCACCCATT	
Cyclophilin	F: CATACAGGTCCTGGCATCTTGTC	56
	R: TGCCATCCAACCACTCAGTCT	
GAPDH	F: TCCGTTGTGGATCTGACCTG	55
	R: TGCTTCACCACCTTCTTGATCTC	
THOC1	F: CCATTGAACAGGCAGACCCT	54
	R: GGCTTCTCCGTGCTAACAGT	
Tie 2	F: CAGCAGACCTCGGAGGCAGGA	58
	R: TGCCCTCTTCAGCTGCAGCATG	
VEGFA	F: GGATGTCTACCAGCGCAGC	56
	R: TCTGGGTACTCCTGGAAGATGTC	
VEGFR1	F: TGGATTTCAGGTGAGCTTGGA	52
	R: TCACCGTGCAAGACAGCTTC	

F, forward; R, reverse; *T*_A_, annealing temperature; CHD11, cadherin-11; GAPDH, glyceraldehyde 3-phosphate dehydrogenase; THOC1, THO complex subunit 1; VEGFA, vascular endothelial growth factor A; VEGFR1, vascular endothelial growth factor receptor 1.

### Microarray of fetal kidneys

First, genomic DNA (gDNA) was extracted from whole sheep blood using the Wizard Genomic DNA Purification kit (Promega, Southampton, UK) as per manufacturer's instructions, then labeled, hybridized to the Affymetrix Human U133 + 2 array (Affymetrix, Santa Clara, CA, USA), and scanned, and gDNA cell intensity files (.cel files) were generated. Probe pairs were identified in which the perfect match (PM) probe has a gDNA hybridization intensity greater than the user-defined threshold and were selected using a .cel file parser script (http://affymetrix.arabidopsis.info/xspecies/), which produces a probe mask file (.cdf) compatible with a range of microarray analysis packages (*e.g.*, Genespring; Agilent Technologies). Probe mask files (.cdf) were produced using gDNA hybridization intensity thresholds ranging from 0 to 1000, as described previously (50). For microarray of fetal kidneys at 65 d gestation, *n* = 8 individual male fetuses from each treatment group (CP and LP) were used, as males were previously identified as particularly susceptible to maternal LP diet (23), and restricting the dataset to one sex would reduce heterogeneity and improve statistical associations. Total RNA was isolated from all samples (Trizol; Life Technologies, Grand Island, NY, USA), and yield and purity were determined prior to microarray using an Agilent 2100 bioanalyzer. RNA (1 μg) was used to generate first-strand cDNA by reverse transcription, followed by synthesis of second-strand cDNA. Double-stranded cDNA products were purified and *in vitro* transcribed to generate biotinylated complementary RNAs (cRNAs). The cRNAs were purified and randomly fragmented before being hybridized to the Affymetrix Human U133 + 2 GeneChip array and stained with streptavidin-phycoerythrin. Arrays were scanned, and .cel raw data files were generated. The DNA and RNA .cel files and the probe mask .cdf files are available to download from the NASC Xspecies website (http://affymetrix.arabidopsis.info/xspecies/).

### Bioinformatic analyses of microarray data

Successfully hybridized genes to the X-species microarray were first normalized using the Li-Wong procedure (51). Differentially expressed probe sets were then filtered on the expression level (*i.e.*, >20–100 percentile) to remove background noise and probes that do not hybridize to the X-species microarray. The *P* value was set at <0.05 and fold change at 1.5-fold to identify probe sets that were significantly up- or down-regulated between treatment groups. The Database for Annotation, Visualization, and Integrated Discovery (DAVID) bioinformatics resource (U.S. National Institute of Allergy and Infectious Diseases, Bethesda, MD, USA; http://david.abcc.ncifcrf.gov/) was then used to identify gene ontologies that were overrepresented in the lists of probe sets, when compared against the list of probe sets expressed above background levels. All bioinformatic data handling was performed using Genespring GX 7.3 (Agilent Technologies).

### Statistical analysis

All data were analyzed using a general linear model for the fixed effects of diet (*e.g.*, CP *vs.* LP) or, where appropriate, the additional effects of sex (male *vs.* female) or time (analyzed as a repeated measure within individuals). For twins, the reduced interindividual variance associated with being nested within a ewe was analyzed as a general linear mixed model, with the ewe included as a random effect (Genstat 15; VSN International, Hemel Hempstead, UK). The suitability of using a general linear model was assessed by generating a histogram of residuals and further residual (on the *y* axis) plots of fitted values and expected normal quantiles. Skewed plots (residual errors) necessitated log_10_ transformation before analysis. Data are presented as means with estimated sem to represent the error between comparisons. Here 95% confidence intervals may be approximated from the normally distributed data as the mean ± 2 se. Correlations between variables were tested using Pearson's correlation tests. Immunohistochemical data were highly positively skewed and were analyzed by generalized linear regression after log_10_ transformation, fitting a γ distribution to residual errors. Values of *P* < 0.05 were accepted as indicating statistical significance.

## RESULTS

### Nutritional status of ewes fed LP diet

**Figure 1.**
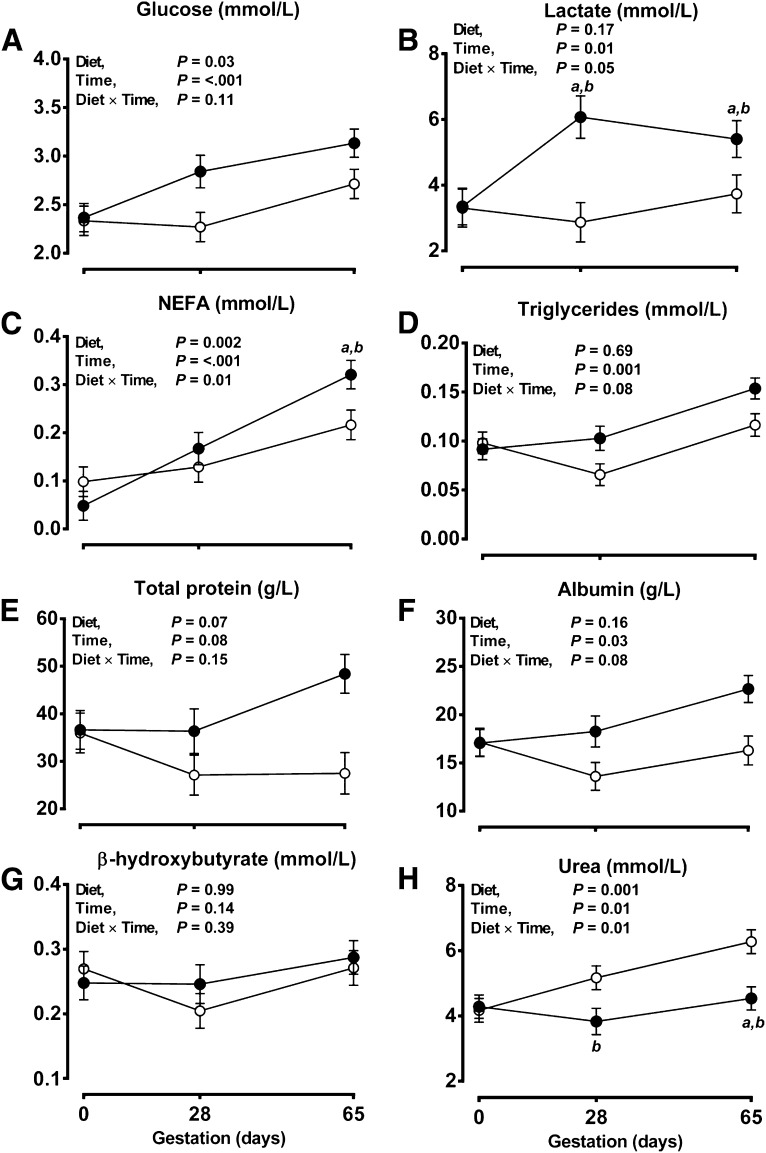
Ewes fed LP diet have reduced plasma urea, but other biomarkers of nutritional status are increased. Glucose (*A*), lactate (***A***), NEFA (*C*), triglyceride (*D*), total protein (*E*), albumin (*F*), β-hydroxybutyrate (*G*), and urea (*H*) samples were obtained prior to pregnancy (d 0′) and at d 28 and 65 of gestation and were analyzed on a Randox Rx-Imola bioanalyzer. Data are means ± estimated sem for ewes fed a CP diet (*n*=15; open symbols) or an LP diet (*n*=16; solid symbols) during early gestation (d 0–65, term ∼145 d). Data were analyzed by the general linear model for the fixed effect of diet, with time included as a repeated measure, and the interaction term (diet×time) using Genstat 15. NS, nonsignificant; NEFA, nonesterified fatty acid. ^*a*^*P* < 0.05 *vs*. d 0. ^*b*^*P* < 0.05 *vs*. control group.

Maternal weight and body condition score (BCS) at artificial insemination (d 0 of gestation) were similar between groups (pooled mean for weight, 56.3±0.5 kg; for BCS, 2.5±0.1 U) and did not deviate significantly from this level to d 65 (at d65: CP, weight=61.2±1.1 kg, BCS=2.7±0.1 U; LP, weight=58.9±1.0 kg, BCS=2.5±0.1 U). From similar initial concentrations in maternal plasma at d 0, plasma NEFAs, glucose, and lactate significantly increased, and plasma triglycerides and albumin showed a strong statistical trend to be increased, whereas plasma urea significantly decreased through early gestation in LP relative to CP ewes (**Fig. 1**). Despite a 50% reduction in dietary crude protein, maternal plasma amino acid concentrations did not differ between treatment groups, although some variation with time was noted (**Table 2**). Plasma osmolality also did not differ between groups at d 0 (pooled mean, 303±2 mOsmol/kg H_2_O) but was, on average, significantly increased in LP *vs.* CP ewes from d 28 onward (pooled mean, 323±4 *vs.* 303±4 mOsmol/kg H_2_O, respectively; *P*<0.001).

**Table 2.** Standard amino acid concentrations in plasma of ewes fed CP or LP diet to d 65 (0.44 gestation)

Amino acid	Diet		Time (d gestation)			*P*		
	CP	LP	0	28	65	D	T	D × T
Alanine	344 ± 28.1	388 ± 28.6	378 ± 33.5	432 ± 36.1	288 ± 34.6	NS	<0.05	NS
Glycine	789 ± 73.7	901 ± 77.8	696 ± 86.7	933 ± 98.5	906 ± 92.9	NS	NS	NS
Isoleucine	94.7 ± 5.54	88.6 ± 6.06	117 ± 6.60	88.5 ± 7.75	69.1 ± 6.94	NS	<0.001	NS
Leucine	126 ± 6.90	109 ± 7.00	145 ± 8.20	114 ± 8.80	92.0 ± 8.50	NS	<0.001	NS
Valine	416 ± 26.5	382 ± 28.3	530 ± 31.5	380 ± 35.8	286 ± 33.1	NS	<0.001	NS
Threonine	53.2 ± 8.41	63.6 ± 9.88	76.0 ± 9.98	62.0 ± 12.3	37.2 ± 11.3	NS	NS	NS
Proline	72.9 ± 4.29	68.5 ± 4.82	84.5 ± 5.11	74.8 ± 6.26	52.5 ± 5.32	NS	<0.001	NS
Phenylalanine	69.3 ± 4.75	59.1 ± 5.19	77.5 ± 5.75	61.2 ± 6.64	54.0 ± 5.85	NS	<0.05	NS
Tryptophan	50.4 ± 9.17	60.3 ± 10.8	70.9 ± 12.4	63.5 ± 12.7	31.7 ± 11.7	NS	NS	NS
Tyrosine	49.4 ± 4.87	41.4 ± 5.29	66.7 ± 6.02	40.8 ± 6.64	28.3 ± 6.02	NS	<0.001	NS
Lysine	181 ± 39.1	225 ± 47.3	213 ± 45.9	265 ± 55.4	131 ± 57.5	NS	NS	NS
Asparagine	27.8 ± 2.52	23.8 ± 2.83	34.9 ± 3.10	26.4 ± 3.47	16.3 ± 3.25	NS	<0.001	NS
Aspartic acid	6.73 ± 1.39	6.50 ± 1.56	5.61 ± 1.81	5.20 ± 1.89	9.05 ± 1.73	NS	NS	NS
Glutamic acid	109 ± 14.4	82.3 ± 15.4	96.4 ± 16.0	141 ± 17.6	49.2 ± 20.7	NS	<0.001	NS
Total	2306 ± 210	2600 ± 237	2610 ± 245	2861 ± 279	1889 ± 296	NS	NS	NS

Data are mean ± sem concentrations (μM) for ewes fed a CP (*n*=15) or LP (*n*=16) diet to d 65 gestation. Data were analyzed by the general linear model for the fixed effects of diet (D), time (T), or their interaction (D×T) using Genstat 14. NS, nonsignificant. Values of *P* < 0.05 were considered significant.

### Nutritional status of fetuses exposed to a maternal LP diet

**Figure 2.**
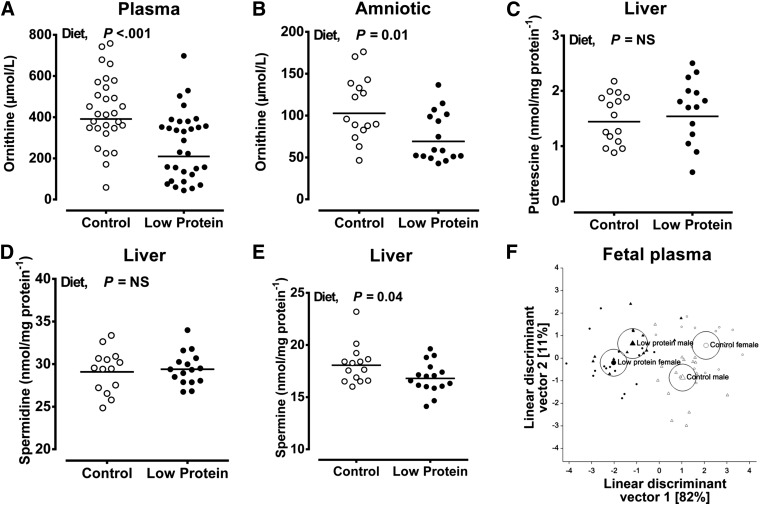
Maternal LP diet reduces the availability of ornithine in the fetal compartment and hepatic spermine concentration. *A*, *B*) Ornithine was measured in fetal plasma (*A*) and amniotic fluid (*B*) using gas chromatography-mass spectrometry (see Materials and Methods). *C–E*) The polyamines putrescine (*C*), spermidine (*D*), and spermine (*E*) were measured in lysates of fetal liver using high-performance liquid chromatography. Data are dot plots of data points from individual fetuses (*A–E*) with the line indicating the mean from each dietary treatment. Data were analyzed by the general linear mixed model for the fixed effects of diet, sex, and their interaction (ewe included as a random effect in the model) using Genstat 15. *F*) Linear discriminant plot generated after multivariate analysis of 18 amino acids in fetal plasma (circles representing 95% confidence interval around group means) conducted after normalization of data (by establishing *z* scores). NS, nonsignificant. Values of *P* < 0.05 were considered significant.

At d 65, neither maternal diet nor fetal sex had any effect on measures of fetal growth (weight and crown-rump length) or on the relative size of fetal organs (**Table 3**). Accordingly, measurements of the major fetal substrates for growth (glucose, lactate, amino acids) were unaltered by maternal diet or fetal sex, and hence sexes are combined (fetal plasma glucose, 0.74±0.09 *vs.* 0.84±0.08 mM; fetal plasma lactate, 4.52±0.21 *vs.* 5.14±0.18 mM; total amino acids, 3900±177 *vs.* 3643±177 μM for CP *vs.* LP, respectively). However, type rather than quantity of micronutrients was subtly altered by maternal diet; for example, fetal plasma glycine was increased by a maternal LP diet (**Table 4**), an effect most apparent in female fetuses (CP male, 450±34 *vs.* LP male 455±31 μM; CP female, 349±31 *vs.* LP female 499±32 μM), whereas the concentrations of proline and asparagine were significantly lower in the plasma of female *vs.* male fetuses, regardless of diet (Table 4). As expected from maternal nutritional status, urea concentration in the amniotic fluid was significantly reduced in LP *vs.* CP fetuses (3497±339 *vs.* 6916±351 μM; *P*<0.001). Due to complex, dependent interrelationships between amino acids in the fetal compartment, we used a multivariate statistical approach to reveal potential treatment effects: linear discriminant analysis of normalized (*z* scores) standard and nonstandard (*e.g.*, ornithine) amino acids indicated a significant separation due to maternal diet (**Fig. 2*F***), largely contributed by the significant reduction in ornithine in LP relative to CP fetuses (Fig. 2*A*, *B*). Further analysis of fetal hepatic polyamine concentrations (the major site for polyamine synthesis in the fetus) indicated a significant reduction in fetal liver spermine, but not putrescine or spermidine, in LP relative to CP fetuses (Fig. 2*C–E*).

**Table 3.** Fetal body and organ weight at d 65 (0.44 gestation) from ewes fed control or low protein diet

Parameter	Male fetuses		Female fetuses		*P*		
	CP	LP	CP	LP	D	S	D × S
*n*	7	8	8	8			
Fetal weight (g)	126 ± 8	123 ± 8	131 ± 9	120 ± 8	0.42	0.90	0.68
Crown-rump length (cm)	16.6 ± 0.4	17.4 ± 0.4	17.3 ± 0.4	16.7 ± 0.4	0.74	0.88	0.09
Heart weight (g/100 g)	0.95 ± 0.03	0.91 ± 0.03	0.85 ± 0.03	0.97 ± 0.03	0.39	0.56	0.02
Kidney weight (g/100 g)	1.42 ± 0.05	1.42 ± 0.05	1.28 ± 0.06	1.44 ± 0.05	0.23	0.33	0.22
Liver weight (g/100 g)	6.90 ± 0.19	6.61 ± 0.21	6.64 ± 0.22	6.86 ± 0.19	0.85	0.97	0.23

Data are means ± sem for ewes bearing twins fed a CP diet (*n*=15 ewes, *n*=30 fetuses) or an LP diet (*n*=16 ewes, *n*=32 fetuses) to d 65 or 0.44 gestation. Data were analyzed by the general linear mixed model for the fixed effects of diet (D; CP *vs.* LP) or fetal sex (S; male or female). Ewe was incorporated as a random effect in the model due to the nested, reduced variability of twins sharing the same intrauterine environment. All analyses were conducted using Genstat 14, and statistical significance was considered acceptable at *P* < 0.05; effects on fetal organs were considered only after correction for fetal body size. Maternal weights at postmortem did not differ between diet groups (CP, 60.5±1.0 *vs.* LP, 58.4±1.0 kg; *P*=0.15).

**Table 4.** Standard measureable amino acid concentrations (μm) in fetal plasma at 65 d (0.44 gestation)

Amino acid	Diet		Sex		*P*		
	CP	LP	M	F	D	S	D × S
Alanine	285 ± 14.5	266 ± 14.5	280 ± 14.7	272 ± 14.2	NS	NS	NS
Glycine	397 ± 22.8	479 ± 26.2	454 ± 24.3	426 ± 24.3	0.01	NS	0.03^*a*^
Isoleucine	78.9 ± 4.44	76.1 ± 4.11	82.8 ± 4.48	72.5 ± 4.33	NS	NS	NS
Leucine	140 ± 6.06	127 ± 5.93	141 ± 6.06	126 ± 5.96	NS	NS	NS
Valine	212 ± 12.3	199 ± 11.3	216 ± 12.3	196 ± 11.7	NS	NS	NS
Serine	700 ± 49.1	691 ± 48.1	678 ± 47.3	717 ± 50.0	NS	NS	NS
Threonine	448 ± 35.3	463 ± 32.9	498 ± 34.6	417 ± 33.5	NS	NS	NS
Proline	119 ± 5.99	116 ± 5.57	127 ± 5.99	109 ± 5.79	NS	<0.05	NS
Phenylalanine	175 ± 8.97	174 ± 8.75	187 ± 9.14	163 ± 8.99	NS	NS	NS
Tryptophan	87.2 ± 12.7	88.6 ± 12.7	92.0 ± 11.3	84.0 ± 11.0	NS	NS	NS
Tyrosine	277 ± 27.7	231 ± 22.9	269 ± 23.1	241 ± 22.4	NS	NS	NS
Lysine	702 ± 65.1	540 ± 61.8	662 ± 63.9	574 ± 62.8	NS	NS	NS
Asparagine	54.6 ± 2.87	54.1 ± 2.73	59.6 ± 2.77	49.4 ± 2.77	NS	<0.05	NS
Aspartic acid	9.66 ± 0.66	9.16 ± 0.60	10.4 ± 0.66	8.41 ± 0.66	NS	NS	NS
Glutamic acid	218 ± 16.8	224 ± 15.9	233 ± 16.3	209 ± 16.5	NS	NS	NS
Total	3821 ± 179	3580 ± 170	3832 ± 174	3557 ± 174	NS	NS	NS

Data are mean ± sem concentrations (μM) for fetuses of ewes fed a CP diet (*n*=29: males, *n*=13; females, *n*=16) or an LP diet (*n*=31: males, *n*=16; females, *n*=15) to d 65 gestation. Data were analyzed by the general linear mixed model for the fixed effects of diet (D), sex (S), or their interaction (D×S) using Genstat 14. The ewe was included as a random term in the model. NS, nonsignificant. Values of *P* < 0.05 were considered significant.

a Data illustrating the statistically significant interaction are in the Results section.

### Fetal kidney transcriptome and macro- and microvascular development after exposure to a maternal LP diet

**Figure 3.**
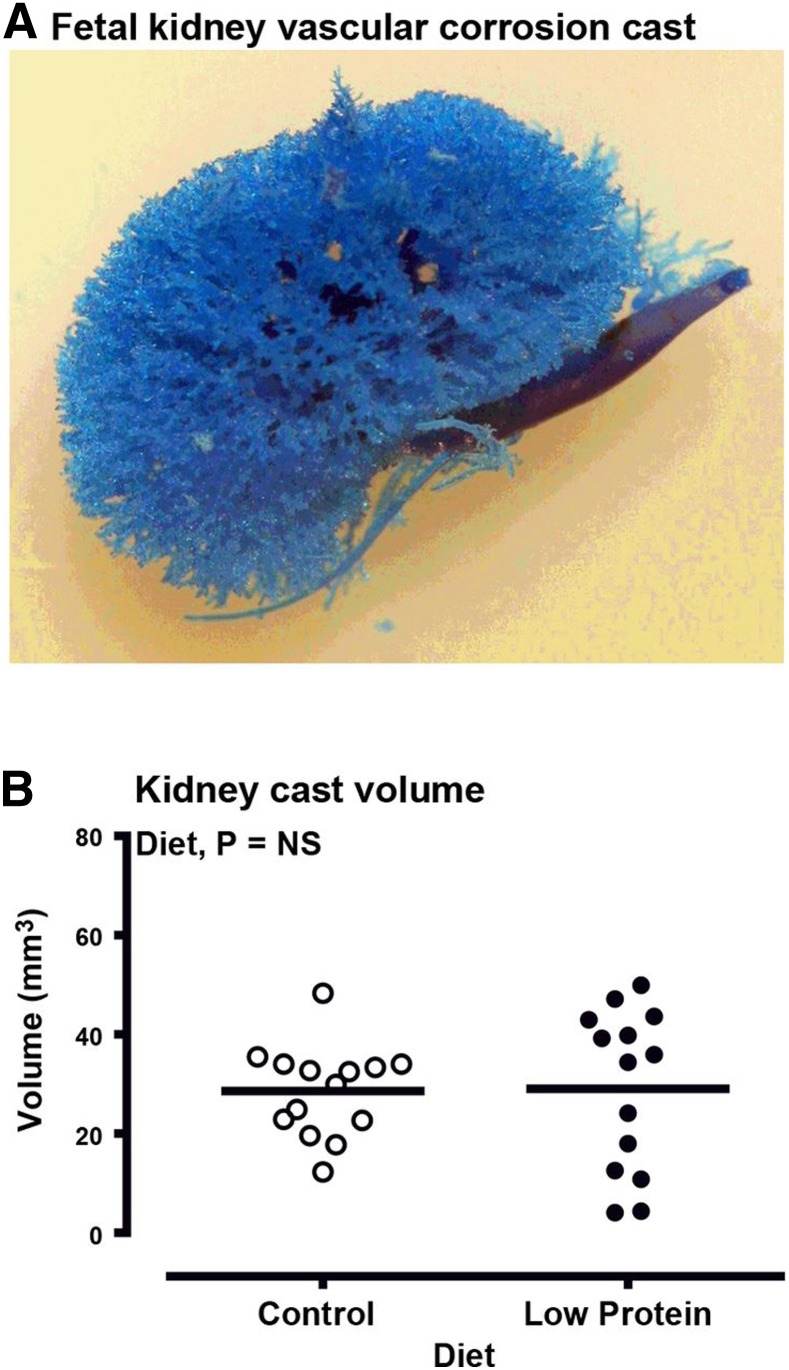
Fetal kidney macrovasculature is unaffected by maternal LP diet. *A*) Representative vascular corrosion cast of a fetal kidney generated using Batson's No. 17 Plastic Replica and Corrosion Kit. Volume and surface area of resin casts was quantified using a Nanotom high-resolution micro-CT scanner. *B*) Dot plot of quantified volumetric data from casts, line at predicted mean for each group. Data are for ewes fed a CP diet (control fetuses: male, *n*=8; female, *n*=7) or LP diet from d 0 to 65 of gestation (LP-exposed fetuses: male, *n*=8; female, *n*=8). Data were analyzed by the general linear mixed model for the fixed effects of diet, sex, and their interaction term (diet×sex) using Genstat 15. Supplemental Movie S1 illustrates 3D volumetric acquisition of vascular corrosion cast data. NS, nonsignificant. Values of *P* < 0.05 were considered significant.

**Figure 4.**
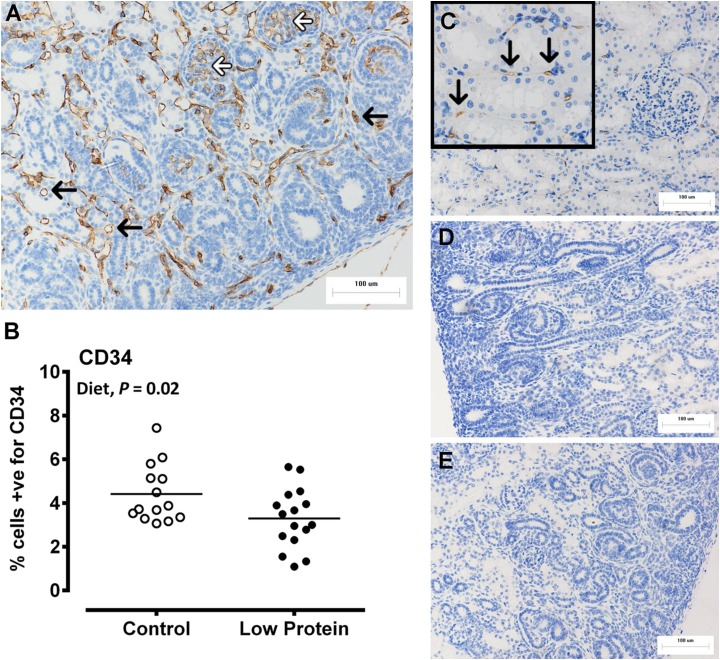
Fetal kidney microvasculature (density of endothelial cells; CD34) is blunted by maternal low protein diet. Fetal kidney microvasculature was indirectly determined using immunohistochemistry and quantification of cells positive for CD34. *A*) Representative micrograph of the nephrogenic zone, CD34 staining is brown, black arrows indicate peritubular capillaries, white arrows show staining of glomerular capillaries. *B*) Dot plot of individual data points for control and LP-exposed fetuses (control: male, *n*=8; females *n*=7; LP diet: male, *n*=8; female, *n*=8). *C*) Positive control, adult ovine kidney. *D*, *E*) Negative controls, omission of primary antibody (*D*) and rabbit IgG (*E*). All images are ×200. Data were analyzed by the general linear mixed model for the fixed effects of diet, sex, and their interaction term (diet×sex) using Genstat 15. NS, nonsignificant. Values of *P* < 0.05 were considered significant.

**Figure 5.**
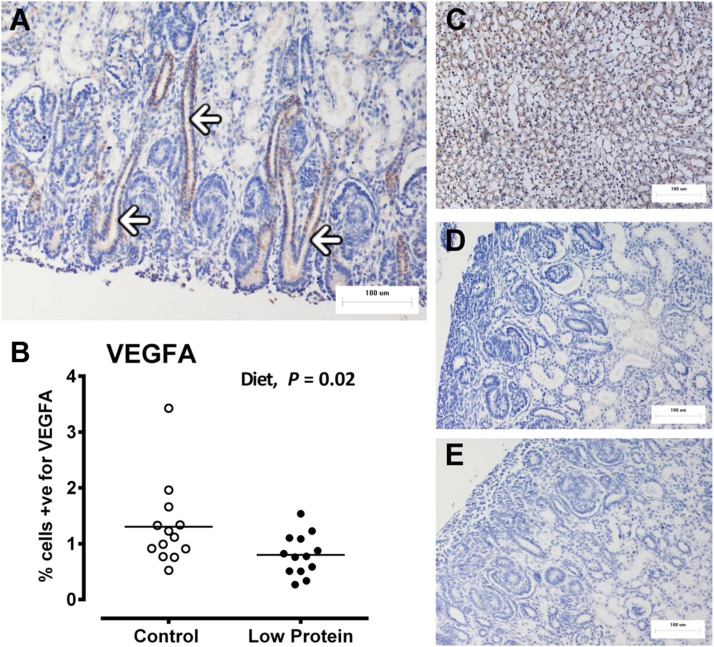
Fetal kidney angiogenic potential, measured as VEGFA, is blunted by maternal LP diet. Fetal kidney angiogenic potential was indirectly determined using immunohistochemistry and quantification of cells positive for VEGFA. *A*) Representative micrograph of the nephrogenic zone: VEGFA staining is brown; white arrows indicate developing collecting ducts. *B*) Dot plot of individual data points for control and LP-exposed fetuses (control: male, *n*=8; females *n*=7; LP diet: male, *n*=8; female, *n*=8). *C*) Positive control, mouse kidney. *D*, *E*) Negative controls, omission of primary antibody (*D*) and rabbit IgG isotype control (*E*). All micrographs were taken at ×200. Data were analyzed by the general linear mixed model for the fixed effects of diet, sex, and their interaction term (diet×sex) using Genstat 15. NS, nonsignificant. Values of *P* < 0.05 were considered significant.

The macrovascular structure of the fetal kidneys was directly assessed using vascular corrosion casting, which produced a detailed 3D representation of the major vessels in the fetal kidneys at 65 d gestation (**Fig. 3*A***). Micro-computed tomography (micro-CT) of corrosion casts indicated no effect of maternal diet nor fetal sex on cast volume (pooled sexes, Fig. 3*B*) or surface area (882±156 *vs.* 1069±135 mm^2^ for control *vs.* low protein, respectively; *P*=0.40), but each was highly correlated (*R*^2^=0.97; *P*<0.001). In contrast, the microvascular density of the fetal kidneys (assessed indirectly by IHC staining of CD34 and VEGFA) was significantly blunted in LP-exposed *vs.* CP fetuses (CD34, **Fig. 4**; VEGFA, **Fig. 5**), particularly in the nephrogenic zone of the kidney. Analysis at the transcript level (qPCR) complemented the protein data with respect to blunted microvascular developmental potential in the kidney of LP-exposed fetuses (**Table 5**).

**Table 5.** Blunted angiogenic potential in the kidney of fetuses exposed to maternal LP diet, as revealed by transcript expression of angiogenic factors

Gene	Diet		Sex		*P*		
	CP	LP	M	F	D	S	D × S
*Angiopoietin*	13.7 ± 0.79	11.5 ± 0.74	13.1 ± 0.74	11.9 ± 0.79	0.062	NS	NS
*Tie 2*	1.52 ± 0.08	1.32 ± 0.07	1.40 ± 0.07	1.42 ± 0.08	0.065	NS	NS
*VEGFA*	13.7 ± 0.63	11.4 ± 0.59	12.8 ± 0.59	12.0 ± 0.63	0.014	NS	NS
*VEGFR1*	16.7 ± 0.99	13.7 ± 0.92	14.4 ± 0.92	15.6 ± 0.99	0.036	NS	NS

Data are mean ± sem units × 10^−2^ for fetuses from ewes fed a CP diet (*n*=15) or an LP diet *n*=16) to d 65 gestation. Data were analyzed by the general linear mixed model for the fixed effects of diet (D), sex (S), or their interaction (D×S) using Genstat 14. NS, nonsignificant. Values of *P* < 0.05 were considered significant.

Transcriptomics of the d 65 (0.44 gestation) fetal kidney identified a total of 9218 genes with known accession numbers that successfully hybridized to the X-species microarray. From this background list, after a strict data normalization and filtering process, a total of 52 genes were significantly (*P*<0.05, fold change>1.5) up-regulated and 31 significantly down-regulated (Tables S1 and S2, respectively) in LP *vs.* CP fetuses. The pathways that were significantly overrepresented (*i.e.*, >10% known genes in each pathway) within each up- or down-regulated gene list are given in Table S3; of interest, the majority of pathways to which the up-regulated genes in LP-exposed fetal kidneys corresponded were concerned with negative regulation of cellular processes (*i.e.*, inhibition of cellular growth), and notable genes of interest were Bcl-2-associated transcription factor 1 (*BCLAF1*), *S*-methyl-5′-thioadenosine phosphorylase, and DNA (cytosine-5)-methyltransferase 3A. The majority of pathways that the down-regulated genes in low protein exposed fetal kidneys associated with were positive regulation of biosynthetic processes and cell adhesion, with notable genes of interest being eukaryotic translation initiation factor 5A (*EIF5A*) and cadherin-11 (CDH11). Using qPCR to validate selected microarray results, we confirmed down-regulated expression of THOC1 and CDH11 in LP relative to CP fetuses. Taken together, the fetal kidney microarray data indicated that there is considerable endogenous variation in gene expression between individuals in the same nutritional group, but a maternal LP diet specifically affects the fetal kidney transcriptome at 0.44 d gestation, with the net balance of gene expression in biosynthetic pathways suggesting reduced intracellular availability of metabolic substrate for renal cell development, reduced cell-cell adhesion, and increased apoptosis.

## DISCUSSION

We previously observed maternal PEM during early gestation to specifically affect development of the fetal kidney to reveal an adult phenotype with fewer nephrons, microvascular rarefaction, and blunted kidney function (23). We interpreted these effects as potentially underpinning the relationship between PEM, low birth weight, and adult (particularly renal) health or disease (52). However, due to a lack of available biofluids, we were unable to directly attribute our fetal and subsequent adult phenotype to any specific change in the pattern of nutrients in either the maternal or fetal compartments. Here we repeated our PEM paradigm using a larger cohort of serial sampled ewes to generate sufficient numbers of male and female twin fetuses, spot-sampled at d 65 (0.44 gestation). We hypothesized that maternal PEM diminishes availability of fetal macro- and micronutrients to compromise development of the fetal kidney vasculature, effects that underpin nutritional programming of kidney development. Combining our established ovine model of PEM with state-of-the-art transcriptomic analyses, we “phenocopied” and extended our previous data of blunted developmental potential, particularly microvascular, in the fetal kidney. We now extend these data to show, for the first time, that this effect is most probably mediated *via* diminished activity of the ornithine cycle, reducing availability of substrate for polyamine biosynthesis to fuel microvascular cell development. Equally, we rule out any effects of maternal or fetal macronutrient balance on fetal kidney macrovascular development. We also failed to observe any sex-specific response to maternal PEM. As a consequence of maternal PEM, defective fetal amino acid/polyamine metabolism most probably underpins programming of fetal kidney development and subsequent function.

First, and notably, we confirm our previous observations on the effect of PEM on the fetal kidney: reduced transcript expression and protein abundance of the key drivers of angiogenesis (VEGFA, VEGFR1, Ang1, and Tie 2; Table 4 and Fig. 5), were evident, together with reduced abundance of CD34^+^ cells (an endothelial cell marker, Fig. 4). These effects were greatest in the nephrogenic zone (for angiogenic factors) and peritubular capillaries and mature glomeruli (for CD34, as observed in human fetal kidneys) (53). Similar effects in the fetal pancreas (reduced VEGFA and microvascular density, increased apoptosis) have been observed previously after maternal PEM in a rat model (54, 55). Hence, we suggest that a common response to maternal PEM is reduced developmental potential of organs (our focus is the kidney) through a reduction of factors that support cell growth (*e.g.*, angiogenesis, vasculogenesis) and an increase in factors that inhibit growth (*e.g.*, apoptosis). An unbiased global transcriptomic overview of differences in fetal kidney gene expression between control and PEM supports this view (Supplemental Tables S1–S3). The question arises as to what extent reduced microvascular development, in part engendered by inadequate organ perfusion, *i.e.*, reduced regional macrovascular development, can physically restrict nutrient supply to developing organs.

To address this question, we generated a macrovascular corrosion cast of the fetal kidneys at d 65 gestation (Supplemental Movie S1 and Fig. 3) and quantified the cast volumetrically by micro-CT. Batson's methylmethacrylate, as used here, is the preferred resin for such studies (56), but using this technique, we observed no effect of PEM on intrarenal macrovascular structure or density between treatment groups. We therefore infer from these data that the ability to supply the kidneys with sufficient blood to support growth is not compromised by maternal PEM. Hence, when taken together with reduced microvascular density (Fig. 4), we can infer that maternal PEM has an effect on the fetal kidney at a cellular level (*e.g.*, endothelium) to either reduce the ability to functionally enable growth (*e.g.*, by transfer of sufficient substrate) or physically support growth (*e.g.*, reduced cell adhesion). It should be noted, however, that using the corrosion casting technique has some drawbacks: while we infused sufficient resin (7 ml) in a retrograde fashion into the umbilical artery to perfuse the intact fetal body and observe reverse perfusion of the umbilical vein, we could not witness perfusion of the fetal kidneys. Thus, while vascular corrosion casting with micro-CT quantification has been used previously to image individual renal corpuscles in adult animals (57), but not to our knowledge in fetuses, our macrovascular data may have underestimated the true vascularity of the kidney. Nevertheless, we now provide robust evidence that maternal PEM has a significant effect on the developing fetal kidney at a cellular level, effects that can lead to an anatomically and physiologically compromised mature adult kidney. This then raises the question as to the extent by which these subtle effects of maternal PEM in the fetal kidney associate with specific changes to fetal macro- and micronutrient balance. Indeed, can we infer that fetal PEM is a consistent response to maternal PEM?

For the first 65 d of pregnancy in sheep, *ca.* 50% protein restriction had few obvious effects on the mother, such as reduced weight gain, or on the levels of intermediary metabolites in her circulation. Significantly reduced plasma urea was noted, but levels remained within published reference ranges for sheep (58, 59). Essentially, the dietary regimen was asymptomatic vis-à-vis maternal weight gain, body composition and plasma chemistry. Serial sampling, however, revealed a trend with time in the LP-fed ewes from which we may speculate a metabolic effect, an increased reliance on lipolysis for energy; and a physiological effect, a reduced renal ability to concentrate urine and, hence, greater free water clearance. The latter effect is inferred by a concomitant early increase (*cf.* control ewes) in glucose, lactate, total protein, and albumin together with an increase in plasma osmolality. In rats, PEM reduced plasma volume expansion (60). Feeding of an LP diet is known to reduce plasma urea (61, 62), which can have a diuretic effect as the renal medullary interstitial gradient for fluid reabsorption is reduced (63). Urea is a very small molecule that can readily cross the placenta (64). The urea/ornithine cycle is an important metabolic pathway generating valuable intermediary metabolites for the growing fetus. We therefore asked whether these maternal effects of an LP diet had significance for the fetus, primarily in terms of the growth and development of the kidney.

For most macro- and micronutrients, the relationship between maternal and fetal compartments is not linear, because the placenta considerably consumes or produces substrate to protect the fetal nutritional milieu (65). Placentation in sheep begins around d 28 and is established by d 65 (66). Previous studies have illustrated that maternal nutrient deprivation can affect the fetal nutritional environment, but considerable study-to-study variability exists, and a consistent response has not emerged (29–32, 34). Here we show in a large group of fetuses of both sexes that at around 0.40 gestation in the sheep, maternal PEM has little effect on fetal macronutrient status. There was no difference in fetal plasma glucose, lactate, or total amino acid (Table 3) concentrations: the primary sources of energy for fetal oxidative metabolism and growth (67, 68). Consequently, growth of the fetus to this stage (whether a male or female) was also unaffected by maternal diet. However, notably, closer statistical examination using multivariate discriminant analysis of the standardized variation in measureable amino acids reveals a consistent pattern in male and female LP-exposed *vs.* CP fetuses (Fig. 2*F*) that can be largely explained by the change in only a few amino acids and their intermediary metabolites. Our principal observations (*i.e.*, the variates with the largest discriminant scores), which were consistent in both fetal plasma and amniotic fluids, was that urea and ornithine were significantly reduced and glycine significantly increased in LP *vs.* CP fetuses. Similar effects, albeit in different nutritional paradigms, have been observed previously in sheep (29, 33) and nonhuman primates (34).

The ability of maternal PEM to specifically affect fetal urea/ornithine and glycine metabolism is of interest, particularly with regard to reduced developmental potential in the fetal kidney. Ornithine is the primary precursor for the synthesis of the polyamines putrescine, spermidine, and spermine (69). Polyamines have diverse biological roles in the cell but primarily fuel proliferation and differentiation (70), and when they are depleted in mammalian cells, growth ceases (71). In the livers of fetuses exposed to maternal PEM, we detected significantly less spermine (Fig. 2*E*). The liver is the primary site for polyamine synthesis in the fetus and is a better index of fetal polyamine status, as opposed to values in fetal plasma or amniotic fluid (29). Indeed, in our hands, polyamines were undetectable in amniotic fluid (assay sensitivity of 0.1 nM). Interestingly, our unbiased transcriptomic screen identified a significant down-regulation of eukaryotic initiation factor 5A (*EIF5a*), a gene essential for translation-elongation and hence protein synthesis (72). EIF5A is the only known protein to contain hypusine, a nonclassical amino acid formed from spermidine (73). Moreover, glycine and serine are readily interconvertible, but serine to glycine is favored in highly proliferative cells (such as a developing fetus) due to the net formation of 5-methyltetrahydrofolate (5-meTHF), which is used to support a number of cellular reactions, including nucleotide synthesis and DNA methylation. Increased glycine concentration, as observed in our study in the fetal compartment, through either increased dietary supply or blockade of the glycine cleavage system, can deplete 5-meTHF and inhibit proliferation of endothelial cells (74), an effect witnessed in this and previous studies by us (23), in the kidneys of PEM fetuses (less CD34^+^ staining). In addition, decreased cellular polyamines can influence the ability of cells to participate in DNA methylation reactions (75). This raises the possibility that maternal PEM may act, *via* a blunting of the fetal ornithine cycle and concomitant knock-on effects on cellular polyamine metabolism, to alter the epigenetic status of developmentally important genes. A consequence, as witnessed here, is altered organ development that persists and manifests as longer-term organ dysfunction (15, 23).

In summary, we illustrate how mild, maternal PEM experienced during early gestation can significantly restrict the developmental potential of the fetal kidney and highlight a nutritional pathway that may underpin this effect. Maternal PEM reduces maternal and fetal urea concentrations to constrain the fetal ornithine cycle and reduce availability of substrates (*e.g.*, polyamines) for cell proliferation. Increased glycine in the fetal compartment may also represent depleted micronutrient availability (*e.g.*, 5-meTHF) that exacerbates the balance of nutrients to favor cellular arrest rather than growth.

## Supplementary Material

Supplemental Data
