# Supplementary material for: Maternal protein-energy malnutrition during early pregnancy in sheep impacts the fetal ornithine cycle to reduce fetal kidney microvascular development
Source: FASEB J. 2014 Nov;28(11):4880–92. doi: 10.1096/fj.14-255364 (PMC4216596; doi:10.1096/fj.14-255364)
Supplement: Supplemental Data [file supp_28_11_4880__index.html]

Maternal protein-energy malnutrition during early pregnancy in sheep impacts the fetal ornithine cycle to reduce fetal kidney microvascular development — Maternal protein-energy malnutrition during early pregnancy in sheep impacts the fetal ornithine cycle to reduce fetal kidney microvascular development — Supplemental Data 

# Maternal protein-energy malnutrition during early pregnancy in sheep impacts the fetal ornithine cycle to reduce fetal kidney microvascular development

## Supplemental Data

**Files in this Data Supplement:**

- Supplemental Data - (*14-255364SuppData.zip; compressed file 6.31 MB*)
