# Supplementary material for: Maternal protein-energy malnutrition during early pregnancy in sheep impacts the fetal ornithine cycle to reduce fetal kidney microvascular development
Source: FASEB J. 2014 Nov;28(11):4880–92. doi: 10.1096/fj.14-255364 (PMC4216596; doi:10.1096/fj.14-255364)
Supplement: Supplemental Data [file supp_fj.14-255364_14-255364SuppData.zip › 14-255364SuppData.docx]

**Supplementary Information.**

**Movie file: Attached is a file illustrating how 3D volumetric data on fetal macrovascular structure was obtained using µCT. Title: “Fetal Kidney Macrovascular Structure”**

**Table S1. Significantly up-regulated genes in the low protein exposed fetal kidney at 0.44 dGA**

| **Gene ID** | **Probeset ID** | **Fold-change** | **P-value** |
| --- | --- | --- | --- |
| LAMP2 | 203041_PM_s_at | 13.20 | 0.006 |
| 10orf46 | 227257_PM_s_at | 4.47 | <.001 |
| PRORSD1P | 237291_PM_at | 4.34 | <.001 |
| HECTD1 | 241955_PM_at | 3.64 | <.001 |
| CPEB4 | 224828_PM_at | 2.57 | <.001 |
| JUN | 201465_PM_s_at | 2.55 | <.001 |
| CCNI | 208656_PM_s_at | 2.43 | <.001 |
| FXR1 | 201635_PM_s_at | 2.27 | <.001 |
| TRIM33 | 210266_PM_s_at | 2.20 | <.001 |
| UTP23 | 227402_PM_s_at | 2.17 | <.001 |
| BCLAF1 | 214499_PM_s_at | 2.15 | <.001 |
| LCOR | 228454_PM_at | 2.09 | <.001 |
| ANKRD17 | 225852_PM_at | 2.06 | <.001 |
| MLL5 | 223189_PM_x_at | 2.04 | <.001 |
| XPNPEP1 | 208453_PM_s_at | 2.01 | <.001 |
| RBFOX2 | 213901_PM_x_at | 1.98 | <.001 |
| CSNK2A1 | 212075_PM_s_at | 1.89 | <.001 |
| TRIM62 | 236845_PM_at | 1.89 | 0.038 |
| GALNT1 | 201723_PM_s_at | 1.88 | <.001 |
| HSPA1A | 200800_PM_s_at | 1.85 | 0.003 |
| HNRNPC | 200751_PM_s_at | 1.84 | <.001 |
| DNMT3A | 218457_PM_s_at | 1.84 | <.001 |
| PPIG | 208994_PM_s_at | 1.83 | <.001 |
| MTAP | 216685_PM_s_at | 1.83 | <.001 |
| TCF4 | 212382_PM_at | 1.82 | <.001 |
| SF3B1 | 201070_PM_x_at | 1.82 | 0.003 |
| RPS2 | 203107_PM_x_at | 1.82 | <.001 |
| NAA15 | 219158_PM_s_at | 1.81 | <.001 |
| NOP56 | 200874_PM_s_at | 1.78 | <.001 |
| FLRT2 | 204358_PM_s_at | 1.77 | <.001 |
| AFTPH | 217939_PM_s_at | 1.76 | <.001 |
| SYNCRIP | 209024_PM_s_at | 1.72 | <.001 |
| TRPS1 | 224218_PM_s_at | 1.72 | 0.001 |
| PAK3 | 214607_PM_at | 1.67 | <.001 |
| HS3ST6 | 239547_PM_at | 1.67 | 0.003 |
| CGGBP1 | 206861_PM_s_at | 1.65 | <.001 |
| BBX | 223134_PM_at | 1.64 | <.001 |
| MTF2 | 203346_PM_s_at | 1.64 | 0.002 |
| UBN1 | 209088_PM_s_at | 1.60 | <.001 |
| PRPF40A | 214941_PM_s_at | 1.59 | <.001 |
| RNF7 | 224439_PM_x_at | 1.59 | 0.002 |
| CNOT7 | 218250_PM_s_at | 1.58 | <.001 |
| RBFOX2 | 212104_PM_s_at | 1.56 | 0.004 |
| PGRMC1 | 201120_PM_s_at | 1.55 | <.001 |
| HBXIP | 202300_PM_at | 1.55 | <.001 |
| C5orf24 | 1553107_PM_s_at | 1.54 | 0.003 |
| PLEKHN1 | 224125_PM_at | 1.54 | 0.022 |
| H2AFJ | 225245_PM_x_at | 1.54 | <.001 |
| IRX5 | 210239_PM_at | 1.53 | 0.003 |
| ADAM10 | 202604_PM_x_at | 1.51 | 0.003 |
| CCT7 | 200812_PM_at | 1.51 | <.001 |

**Table S2. Significantly down-regulated genes in the low protein exposed fetal kidney at 0.44 dGA**

| **Gene ID** | **Probeset ID** | **Fold-change** | **P-value** |
| --- | --- | --- | --- |
| MT1F | 217165_PM_x_at | -16.98 | 0.041 |
| ZNF41 | 1553216_PM_at | -12.98 | 0.004 |
| UBR3 | 234982_PM_at | -3.36 | <.001 |
| RPL37A | 201429_PM_s_at | -2.69 | 0.004 |
| CNN3 | 201445_PM_at | -2.64 | 0.007 |
| EIF5A | 213753_PM_x_at | -2.60 | 0.012 |
| CDH11 | 207172_PM_s_at | -2.52 | <.001 |
| EIF4G3 | 201936_PM_s_at | -2.47 | <.001 |
| APC | 203525_PM_s_at | -2.38 | <.001 |
| FOXO6 | 239657_PM_x_at | -2.37 | 0.009 |
| PAX2 | 206228_PM_at | -2.33 | <.001 |
| SUGP1 | 215004_PM_s_at | -1.96 | 0.005 |
| MMP16 | 223614_PM_at | -1.83 | <.001 |
| VCP | 208649_PM_s_at | -1.82 | 0.017 |
| OR7E104P | 1566956_PM_at | -1.79 | 0.037 |
| LOC100287017 | 229687_PM_s_at | -1.78 | <.001 |
| LUZP6 | 224656_PM_s_at | -1.77 | <.001 |
| TAF15 | 202840_PM_at | -1.69 | 0.001 |
| INHBA | 227140_PM_at | -1.67 | 0.007 |
| APP | 214953_PM_s_at | -1.63 | <.001 |
| B9D1 | 210534_PM_s_at | -1.63 | <.001 |
| ITM2B | 217732_PM_s_at | -1.61 | 0.004 |
| MARK3 | 202568_PM_s_at | -1.61 | 0.002 |
| HIPK2 | 219028_PM_at | -1.59 | <.001 |
| LOC440434 | 214107_PM_x_at | -1.58 | 0.019 |
| COL3A1 | 201852_PM_x_at | -1.56 | 0.010 |
| NFIC | 213298_PM_at | -1.55 | 0.004 |
| THOC1 | 204064_PM_at | -1.53 | 0.020 |
| TM9SF3 | 222399_PM_s_at | -1.53 | <.001 |
| TFG | 217839_PM_at | -1.53 | 0.005 |
| HIC1 | 230218_PM_at | -1.52 | 0.040 |

**Table S3. Gene ontologies significantly over-represented by up and down-regulated probesets in the fetal kidney at 0.44 dGA**

| **Metabolic process** | **Number of Probes** | **Percentage of total** | **P-value** |
| --- | --- | --- | --- |
| ***Significantly up-regulated genes*** |  |  |  |
| regulation of transcription | 12 | 24 | 0.050 |
| RNA processing | 7 | 14 | 0.011 |
| negative regulation of macromolecule metabolic process | 7 | 14 | 0.031 |
| RNA splicing | 6 | 12 | 0.003 |
| mRNA metabolic process | 6 | 12 | 0.008 |
| negative regulation of transcription | 6 | 12 | 0.017 |
| negative regulation of gene expression | 6 | 12 | 0.024 |
| negative regulation of nucleobase, nucleoside, nucleotide and nucleic acid metabolic process | 6 | 12 | 0.025 |
| negative regulation of nitrogen compound metabolic process | 6 | 12 | 0.028 |
| negative regulation of macromolecule biosynthetic process | 6 | 12 | 0.028 |
| negative regulation of cellular biosynthetic process | 6 | 12 | 0.035 |
| negative regulation of biosynthetic process | 6 | 12 | 0.037 |
| mRNA processing | 5 | 10 | 0.023 |
| ***Significantly down-regulated genes*** |  |  |  |
| positive regulation of macromolecule metabolic process | 6 | 19.4 | 0.016 |
| positive regulation of apoptosis | 5 | 16.1 | 0.007 |
| positive regulation of macromolecule biosynthetic process | 5 | 16.1 | 0.028 |
| positive regulation of cellular biosynthetic process | 5 | 16.1 | 0.032 |
| positive regulation of programmed cell death | 5 | 16.1 | 0.008 |
| positive regulation of transcription from RNA polymerase II promoter | 4 | 12.9 | 0.033 |
| neuron projection morphogenesis | 3 | 9.7 | 0.050 |
| regulation of translation | 3 | 9.7 | 0.034 |
